# Pacemaker-generated stress fracture of the second rib: a case report

**DOI:** 10.1186/s13019-020-01303-y

**Published:** 2020-09-16

**Authors:** Jus Ksela, Mark Racman, Rok Zbacnik, Anze Djordjevic, Matevz Jan

**Affiliations:** 1 Department of Cardiovascular Surgery, University Medical Center Ljubljana, Zaloska 7, 1000 Ljubljana, Slovenia; 2 Clinical Institute of Radiology, University Medical Center Ljubljana, Zaloska 7, 1000 Ljubljana, Slovenia; 3 Department of Cardiac Surgery, University Medical Center Maribor, Ljubljanska 5, 2000 Maribor, Slovenia

**Keywords:** Cardiovascular implantable electronic device, Pocket-related complication, Stress fracture, Case report

## Abstract

**Background:**

Pocket-related complications following the implantation of cardiovascular implantable electronic devices primarily include pocket hematoma, infection, skin erosion or decubitus, device migration, and Twiddler's syndrome, with other pathologies such as nerve impairment or bone lesions being extremely rarely encountered. We report a case of a 20-year old asthenic, non-athlete female patient presenting with a device-generated fracture of the second rib several months after sub-muscular permanent pacemaker implantation due to repeated bilateral pre-pectoral pocket infections.

**Case presentation:**

A 20-year old female patient was readmitted to our institution 9 months following sub-pectoral implantation of a permanent pacemaker, complaining of severe pocket-related pain, which arose spontaneously in the absence of direct trauma, intense physical activity or vigorous coughing, and was associated with normal day-to-day activity. To rule out a pacemaker re-infection, a native computed tomography and a positron emission tomography—computed tomography of the thorax were performed. Both modalities excluded an infection but showed a healing fracture and a focus of enhanced metabolic activity in the anterolateral part of the right second rib, indicating a non-traumatic or stress fracture of the bone. Consequently, a complete extraction of the pulse generator and both leads was performed and the smallest available single-chamber pulse generator with a single atrial electrode was implanted in the sub-fascial, pre-muscular pocket in the now recovered and uninfected left subclavicular region, alleviating patient’s severe pain symptoms and significantly enhancing her quality of life.

**Conclusions:**

In the absence of direct trauma, intense physical activity or vigorous coughing, we assume that in this asthenic girl a normal day-to-day motion of the right shoulder has persistently forced the sub-muscularly placed pulse generator toward thoracic wall, putting increased repetitive pressure force on the underlying bones, finally causing a fatigue stress fracture of the second rib. In asthenic phenotype patients with small thorax and short subclavicular distance, a sub-muscular pacemaker implantation can potentially cause unique and unexpected pocket-related adverse events necessitating advanced diagnostics and timely treatment.

## Background

Pocket-related complications following the implantation of cardiovascular implantable electronic devices (CIEDs), including permanent pacemakers (PMs), cardiac resynchronization therapy devices (CRTs), and implantable cardioverter defibrillators (ICDs), have a reported incidence of 2–7% and primarily comprise of pocket hematoma, infection, skin erosion or decubitus, device migration, and Twiddler's syndrome [1]. Apart from these, other pathologies involving deeper anatomical structures surrounding the pulse generator, such as nerve or muscle impairment or bone lesions, are extremely rare [1, 2]. We present an unusual case of a 20-year old asthenic female patient presenting with a device-generated stress fracture of the second rib several months after sub-muscular (i.e. sub-pectoral) permanent PM implantation.

## Case presentation

**Fig. 1:**
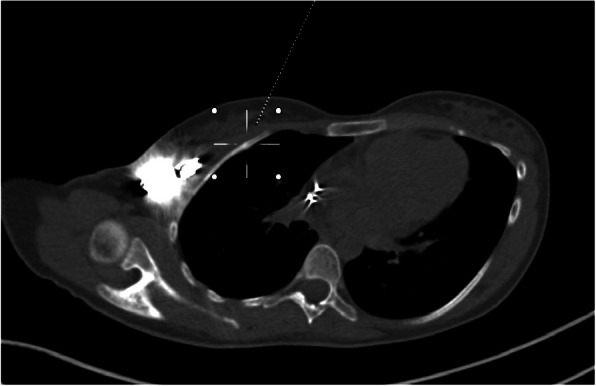
Computed tomography (CT) image in oblique axial plane depicting pacemaker generator and a healing fracture (dotted line) in the anterolateral part of the second right rib

**Fig. 2:**
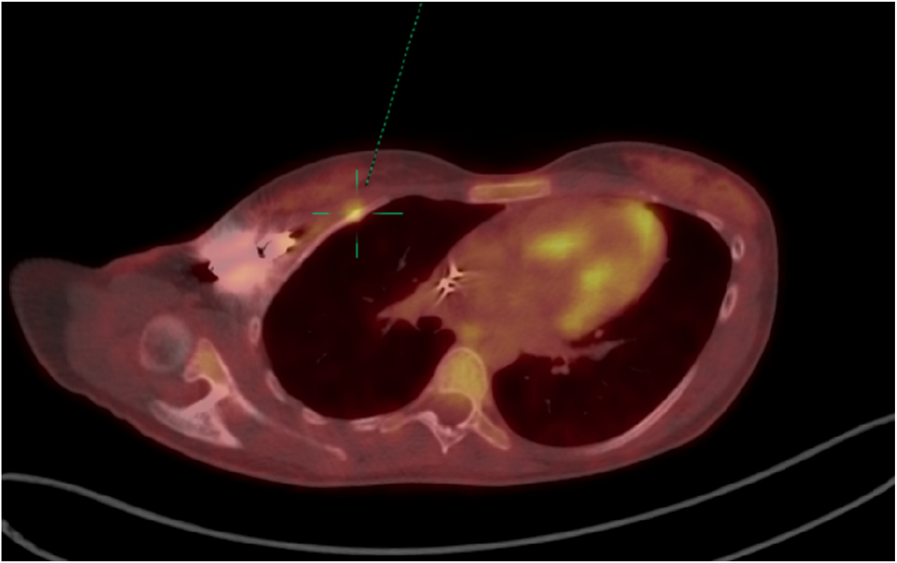
Fusion positron emission tomography CT (PET CT) image in oblique axial plane depicting pacemaker generator and metabolic activity (dotted line) in the healing fracture in the anterolateral part of the second right rib

A 20-year old asthenic, non-athlete Caucasian female was first admitted to our institution for a right-sided pre-pectoral sub-fascial permanent dual chamber PM implantation. Implantation was indicated for sinus node dysfunction resulting from extensive right atrial radiofrequency ablation for treatment of a drug refractory supraventricular tachycardia. Three months later she was readmitted to our hospital due to pocket infection caused by Staphylococcus aureus, thus the extraction of the infected system and reimplantation of a new dual chamber PM in pre-pectoral sub-fascial manner on the contralateral side after discontinuation of antibiotic therapy was indicated. Unfortunately, six months later the patient was readmitted to the department with a left-sided pocket infection again due to Staphylococcus aureus and the extraction of the CIED was indicated once again. Since she refused epicardial PM system implantation, we opted for sub-muscular (i.e. sub-pectoral) implantation of a new dual chamber PM in the now recovered, uninfected and unimpaired right subclavicular region again following the discontinuation of the antibiotic therapy. However, after 9 months she returned to the outpatient clinic complaining of severe right subclavicular pain, which arose spontaneously (firstly as a mild discomfort only two months after PM implantation gradually intensifying over subsequent 7 months to severe levels) in the absence of direct trauma and was associated with normal day-to-day activity performing routine work as a registered nurse in a local hospital. Besides the palpation-induced pain in the right subclavicular region, painful right upper extremity abduction, and slightly elevated body temperature of 37.9 °C, her physical, neurological and endocrinological status (including bone mineral density status assessed by dual-energy X-ray absorptiometry) revealed no significant abnormalities. The chest X-ray reading was normal with appropriate pulse generator and leads position. However, the ultrasound examination of the subclavicular region revealed a small amount of fluid in the PM pocket. To rule out a re-infection of the CIED and/or any lesions of the adjacent tissues, both a native computed tomography (CT) and a positron emission tomography-computed tomography (PET-CT) of the thorax were performed. Both modalities excluded an infection but showed a healing fracture and a focus of enhanced metabolic activity in the anterolateral part of the right second rib (Figs. 1 and 2), indicating a non-traumatic or stress fracture of the bone. After two months of conservative therapy with relative rest and nonsteroidal anti-inflammatory medications no improvement in the patient’s physical performance was observed and thus a complete extraction of the pulse generator and both leads was performed. A new, the smallest available, single-chamber pulse generator with an atrial electrode was implanted in the sub-fascial, pre-muscular pocket in the now recovered and uninfected left subclavicular region. A repeated native CT scan of the upper right hemithorax 6 months after the surgical procedure revealed a healed fracture of the second rib. A year after the final surgery, the patient is clinically stable, managed on an outpatient basis, without any right subclavicular palpation-related pain and with painless full mobility of the right upper extremity.

## Discussion

Pocket-related adverse events following CIED implantation, conventionally divided into acute, sub-acute and late complications, range from more frequently encountered events, such as pocket hematoma, skin decubitus or superficial and deep infection, to less frequently observed events, including CIED dysfunction or material allergy, twisting of the pulse generator inside the pocket (i.e. Twiddler’s syndrome) or migration of the device to the ipsilateral axilla or breast tissue due to improper device fixation to the pectoralis muscle [1]. Complications including injuries to the anterior chest wall structures, such as brachial plexus or sympathetic nerve fibers injury or bone lesions, such as fractures, are extremely rare, especially in the absence of direct trauma [2–4].

Non-traumatic or stress fractures, histologically defined as an imbalance between osteoclast-mediated resorption and osteoblast-mediated repair [5] and pathophysiologically delineated as either insufficiency- or fatigue-generated fracture processes [6], are a relatively common entity across all patient demographics. While insufficiency fractures result from normal loading upon abnormal bone, as encountered in osteoporotic post-menopausal women, fatigue fractures result from an abnormal repetitive load upon normal bone occurring when osteoclast resorption surpasses osteoblast replacement during an abrupt increase in frequency, duration or intensity of activity, especially in young athletes or military recruits [6]. Although stress fractures are predominantly identified as injuries of the weight-bearing bones of lumbar spine, pelvis, and lower extremities, several smaller case series and case reports have also described stress fractures of ribs, primarily in athletes practicing activities generating forceful tensile, compressive and rotational stress on the thorax, such as weight-bearers and lifters, overhead throwers, axial rotators and rowers, and secondarily in non-athlete individuals with endocrinological pathologies or after vigorous coughing or repetitive unphysiological outside pressure [7]. We strongly believe that repetitive unphysiological pressure from the sub-muscularly implanted PM was the most likely pathophysiological mechanism for a stress fracture also in our patient. Namely, in our young, asthenic female patient with no history of intense physical activity, vigorous coughing, any endocrinological disease or direct trauma, a sub-muscularly implanted pulse generator, lying in a discrete subclavicular space directly on the upper ribs, has been persistently forced towards the thoracic wall by a normal day-to-day motion of the right shoulder, putting increased repetitive pressure force on the underlying bones and finally causing a fatigue stress fracture of the second rib 9 months after CIED implantation.

Since stress fractures to the bone are a continuum of mechanical failure ranging from simple bone marrow oedema to small micro-cracks with minor cortical disruption or complete fracture with or without fragments displacement, the most suitable diagnostic procedure is usually location-specific and patient-dependent. Evidence indicates that magnetic resonance imaging (MRI) has the highest combined sensitivity and specificity in the diagnostics of rib stress fractures, outperforming plain radiography, computed tomography (CT), positron emission tomography CT (PET-CT) and bone scintigraphy by delineating rib injury up to 14 days earlier when compared to other diagnostic modalities [8]. However, in patients with CIEDs implanted in either subclavicular region, MRI faces significant interpretation-related disadvantages in readings for rib fractures due to artefacts produced by the metallic housing of the pulse generator, regardless of the implanted system’s MRI-compatibility status. Thus, in CIED patients presenting with unexplained symptoms of painful pocket, CT-based modalities should be regarded as first-line diagnostic tool to enable timely diagnosis and proper treatment. Unsurprisingly, plain radiography reading in our patient was negative and only a CT scan and a PET-CT performed later revealed a stress fracture.

As conservative therapy with partial or complete discontinuation of causative activities is generally advocated to patients with rib stress fracture without fragments dislocation [9], a conservative therapy with relative rest and nonsteroidal anti-inflammatory medications was initially recommended to our patient [10]. Only after no improvement in her physical status with ongoing severe palpation- and arm adduction-generated pain in the right subclavicular region was observed, a surgical extraction of CIED with a de-novo implantation of a single-chamber device on the contralateral side was performed. Afterwards, all pain was alleviated and patient’s quality of life significantly improved. Moreover, bone healing was confirmed on the repeated CT scan 6 months following the surgical intervention. In CIED individuals with exhausted bilateral subclavicular implantation possibilities, an alternative implantation approaches including epicardial leads with sub-xyphoid access and epigastrial supra-muscular device position or leadless transcatheter device implantation directly in the right ventricle can be advised if applicable.

## Conclusions

To conclude, we present, to the best of our knowledge, a so far unpublished clinical scenario of a second right rib stress fracture after sub-pectoral PM implantation in a young asthenic female patient. In the absence of intense physical activity, vigorous coughing, endocrinological pathologies or direct trauma, an increased repetitive pressure force put upon upper ribs by a sub-muscularly implanted pulse generator being persistently forced toward thoracic wall by a normal day-to-day motion of the right shoulder represents the underlying pathophysiological mechanism for fatigue rib stress fracture in this individual. In asthenic phenotype patients with small thorax and short subclavicular distance, a sub-muscular pacemaker implantation can potentially cause unique and unexpected pocket-related adverse events necessitating advanced diagnostics and timely treatment.

## Data Availability

Not applicable.

## Abbreviations

CIED: Cardiovascular implantable electronic device
PM: Pacemaker
CRT: Cardiac resynchronization therapy
ICD: Implantable cardioverter defibrillator
MRI: Magnetic resonance imaging
CT: Computed tomography
PET-CT: Positron emission tomography computed tomography
